# Added value of ophthalmic artery Doppler in prediction of pre‐eclampsia: systematic review and meta‐analysis

**DOI:** 10.1002/uog.70002

**Published:** 2025-08-19

**Authors:** I. Sapantzoglou, P. Antsaklis, V. Pergialiotis, M. I. Chatziioannou, Z. Fasoulakis, M. A. Daskalaki, N. Thomakos, G. Daskalakis, M. Theodora

**Affiliations:** ^1^ First Department of Obstetrics and Gynecology, ‘Alexandra’ General Hospital National and Kapodistrian University of Athens Athens Greece; ^2^ School of Medicine European University of Cyprus Nicosia Cyprus

**Keywords:** Doppler, hypertensive disorders, ophthalmic artery, pre‐eclampsia, prediction, pregnancy

## Abstract

**Objectives:**

Pre‐eclampsia (PE) is a multisystem pregnancy‐specific disorder, and its screening remains a global health priority. The primary goals of this systematic review and meta‐analysis were to accumulate all available data to assess the potential added value of maternal ophthalmic artery (OA) Doppler indices in combination with several established biophysical markers for the prediction of preterm and term PE, and to update the current knowledge regarding the clinical relevance of this biophysical marker.

**Methods:**

This systematic review and meta‐analysis was designed according to the Preferred Reporting Items for Systematic Reviews and Meta‐Analyses (PRISMA) guidelines. MEDLINE, Scopus, ClinicalTrials.gov, EMBASE, Cochrane Central Register of Controlled Trials and Google Scholar databases were searched from inception to 31 January 2025, along with the reference lists of retrieved full‐text papers. We included observational studies that reported on the screening performance of OA Doppler indices for predicting PE, preterm PE (< 37 weeks) and term PE (≥ 37 weeks) (primary outcome). Diagnostic accuracy was assessed using pooled area under the summary receiver‐operating‐characteristics curve (AUC), comparing screening strategies that use maternal factors alone or a combination of maternal factors, uterine artery (UtA) Doppler and mean arterial pressure, with and without the addition of OA Doppler indices.

**Results:**

A total of six peer‐reviewed papers were included in this meta‐analysis, with a total of 10 408 patients. For the outcome of PE, the pooled AUC for screening using maternal factors alone was 0.71 (95% CI, 0.68–0.75) and the pooled AUC for maternal factors with OA Doppler was 0.79 (95% CI, 0.72–0.87). In cases of preterm PE, the pooled AUC for maternal factors plus OA Doppler was 0.88 (95% CI, 0.82–0.94), while the corresponding figure for women who developed term PE was 0.76 (95% CI, 0.65–0.88). The pooled AUC for the combination of maternal factors and UtA pulsatiliy index (PI) in predicting PE was 0.76 (95% CI, 0.66–0.88), while the pooled AUC for maternal factors, UtA‐PI and OA Doppler was 0.85 (95% CI, 0.76–0.94). In cases of preterm PE, the pooled AUC for maternal factors and UtA‐PI was 0.84 (95% CI, 0.78–0.91), while the corresponding figure for maternal factors, UtA‐PI and OA Doppler was 0.88 (95% CI, 0.82–0.93). In cases of term PE, the pooled AUC of maternal factors and UtA‐PI was 0.70 (95% CI, 0.62–0.78), while the corresponding figure for maternal factors, UtA‐PI and OA Doppler was 0.77 (95% CI, 0.70–0.85). The pooled AUC for maternal factors, UtA‐PI and mean arterial pressure (MAP) in predicting PE was 0.80 (95% CI, 0.61–1.06), and the AUC for maternal factors, UtA‐PI and MAP with the addition of OA Doppler was 0.84 (95% CI, 0.61–1.15).

**Conclusions:**

Our study revealed that the addition of OA Doppler to established screening methods may increase the AUC for the outcome of PE and, more precisely, for preterm PE, compared with models not including this marker. However, overlapping 95% CIs limit the robustness and applicability of these results. Since OA assessment is a technically straightforward and precise proxy for the less accessible intracranial circulation, it should be further evaluated by future studies focusing mainly on preterm PE, for which it may be of more value. © 2025 The Author(s). *Ultrasound in Obstetrics & Gynecology* published by John Wiley & Sons Ltd on behalf of International Society of Ultrasound in Obstetrics and Gynecology.

## INTRODUCTION

Pre‐eclampsia (PE) is a multiorgan pregnancy‐specific disorder, with an overall incidence of 5–8% among all pregnancies, and is one of the main causes of maternal morbidity and mortality globally^1^, ^2^. PE screening remains a global health priority, and large‐scale trials have demonstrated the effectiveness of aspirin in the early stages of pregnancy for the prevention of preterm PE^3^. Traditional models for identifying women at high risk for developing PE have focused on risk factors such as maternal characteristics and medical and obstetric history; however, the performance of such models is suboptimal^4^. Recently developed PE prediction models are based on the combination of maternal factors, uterine artery (UtA) Doppler assessment and biochemical markers, demonstrating a significant improvement in the detection of PE compared with traditional screening models^5^.

The traditional hypothesis regarding the pathophysiology of PE is based on underlying placental dysregulation, which is mostly attributed to the inadequate invasion of trophoblasts into the spiral arteries. However, several recent studies that use markers such as reduced cardiac index and increased vascular resistance have suggested alterations to the function of the maternal cardiovascular system preceding the development of PE^6^. This explains the focus of current literature on the investigation of various peripheral vessels as potentially useful biophysical markers for the prediction of PE. The maternal ophthalmic artery (OA) is one of the most studied vessels, given the following convenient attributes: it is the first branch of the internal carotid artery and shares morphological and functional characteristics with the cerebral vasculature^7^, ^8^, ^9^; it is easily accessible for Doppler interrogation; its assessment only requires standard ultrasound equipment; and reference ranges for OA parameters have been established throughout pregnancy^10^.

The primary goal of this systematic review and meta‐analysis was to synthesize all available data to assess the potential incremental value of maternal OA Doppler indices when combined with several established biophysical markers for the prediction of preterm and term PE. We aimed for the results of this meta‐analysis to update the established knowledge regarding the clinical relevance of this novel biophysical marker^10^ and the potential necessity for additional investigation of its utility.

## METHODS

### Information sources and search strategy

MEDLINE, Scopus, ClinicalTrials.gov, EMBASE, Cochrane Central Register of Controlled Trials (CENTRAL) and Google Scholar databases were searched from inception to 31 January 2025, along with the reference lists of electronically retrieved full‐text papers. Our search strategy included the words ‘ophthalmic artery’, ‘Doppler’, ‘prediction’, ‘preeclampsia’, ‘PET’, ‘hypertensive disorders’ and ‘pregnancy’. This systematic review and meta‐analysis was designed according to the Preferred Reporting Items for Systematic Reviews and Meta‐Analyses (PRISMA) guidelines. The study was registered with the PROSPERO international database for systematic reviews (reference number: CRD42023415222).

### Eligibility criteria

The present systematic review included all observational studies (prospective or retrospective cohort, case–control, nested case–control, cross‐sectional) that reported on OA Doppler indices (namely, first peak systolic velocity (PSV1), second peak systolic velocity (PSV2), end‐diastolic velocity (EDV), pulsatility index (PI) and the ratio of PSV2/PSV1 (PR)) and their screening performance for PE. No restrictions were applied based on the trimester in which screening was performed or the type of laboratory assay. The primary outcome of interest was the subsequent development of overall PE, term PE and preterm PE, which were defined according to the gestational age (< 37 or ≥ 37 weeks) at which PE was diagnosed. Case reports, small case series, letters to the editor, animal studies and review articles were excluded. Conference proceedings and abstracts were also excluded, as they lack important information that is necessary for the assessment of study limitations and quality of evidence. No language restrictions were applied.

### Study selection

The process of study selection was conducted in three consecutive stages. First, the titles and abstracts of all electronic papers were reviewed to assess their eligibility. Second, all articles that met or were presumed to meet the eligibility criteria were retrieved in full text. Finally, all observational (both prospective and retrospective) studies reporting on OA Doppler parameters (PSV1, PSV2, EDV, PI or PR) and their screening performance for PE were deemed eligible. Study selection was performed by two authors (I.S., P.A.) independently, while any potential discrepancies were resolved by discussion until consensus was reached. Studies were selected irrespective of the combination of OA Doppler parameters used to predict the development of subsequent PE, as significant heterogeneity was anticipated considering the differences in methods used to detect patients at risk of developing PE, prior to the publication of novel methods.

### Data collection

The following data were extracted from each included study: name of first author, year of publication, study design, study center, study period, timing of assessment, inclusion and exclusion criteria, number of patients, maternal age, prepregnancy body mass index, method of conception, parity, smoking status, ethnicity, prior hypertension, prior diabetes mellitus, and personal and family history of PE. When any of these data were missing, attempts were made to contact the corresponding author. Data extraction was performed by three authors (I.S., P.A., Z.F.), while any disagreements were resolved through their consensus or by discussion with all the authors.

The aim of this study was to assess the added value of OA Doppler indices when combined with clinically established screening methods, including: (1) maternal factors; (2) maternal factors combined with UtA‐PI; and (3) maternal factors, UtA‐PI and mean arterial pressure (MAP).

### Quality assessment

The methodological quality of the included studies were assessed by two independent reviewers (I.S., Z.F.) using the Quality Assessment of Diagnostic Accuracy Studies‐2 (QUADAS‐2) tool^11^, which evaluates the technique of patient selection, the index test, the reference standard for that test and the flow and timing of the test/study. When the two reviewers disagreed, a final decision was made by a third reviewer (M.T.). The overall risk of each study was decided based on the suggestions made by the QUADAS‐2 group^11^.

### Data synthesis

Statistical meta‐analysis was performed using the meta function in RStudio (RStudio Inc., Boston, MA, USA). Statistical heterogeneity was not considered during the evaluation of the appropriate model (fixed‐effects or random‐effects model (REM)) for statistical analysis, as the considerable methodological heterogeneity did not permit the assumption of comparable effect sizes among included studies. A REM was reported eventually as it was considered the most appropriate model. We calculated pooled area under the summary receiver‐operating‐characteristics (SROC) curve (AUC) with 95% CI for the prediction of PE, using precalculated AUCs given in the included studies. Pooled AUCs were retrieved from individual AUCs using the standard metagen function in the meta package in RStudio. To help correct the heterogeneity and the varying sample sizes among included studies, the Hartung–Knapp–Sidik–Jonkman method, instead of the traditional DerSimonian–Laird method, was used for REM analysis. The decision to use this method of analysis was made after considering recent reports that support its superiority over the DerSimonian–Laird model when comparing studies of varying sample sizes and between‐study heterogeneity. Optimal cut‐off values for AUC were considered to define the actual predictive significance of the findings of the meta‐analysis. Specifically, an AUC of < 0.8 was considered of moderate predictive value, an AUC ranging between 0.80 and 0.84 was considered of good predictive value and an AUC of > 0.85 was considered of optimal predictive value. Publication bias was due to be assessed using inspection of retrieved funnel plots for outcomes that included more than 10 studies, as well as using Egger's test, which represents a linear regression analysis that considers the intervention effect estimates and their standard errors, which are weighted by their inverse variance. Considering that this number was not met, determination of publication bias was not performed.

## RESULTS

### Study selection and characteristics

**Figure 1:**
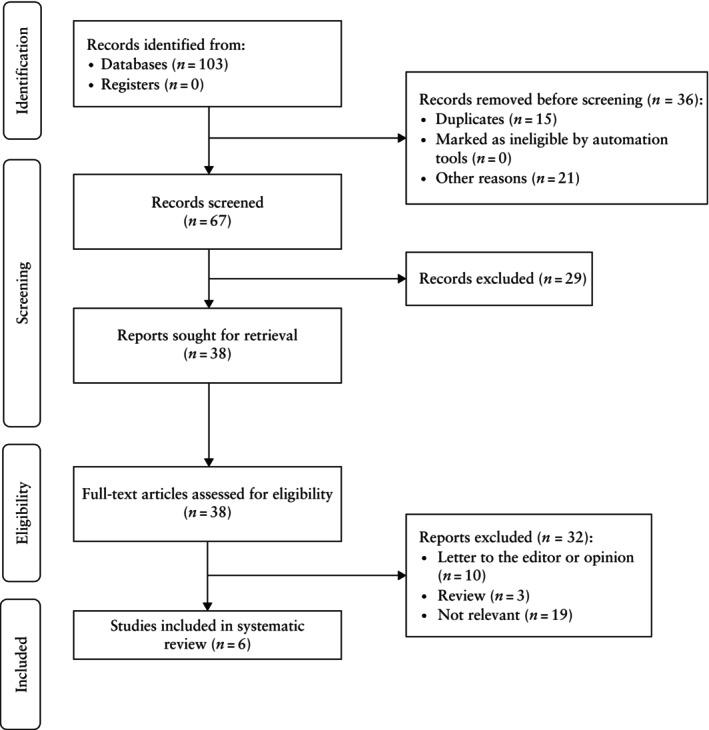
Flowchart summarizing inclusion of studies in systematic review and meta‐analysis.

The search identified 103 potentially relevant studies, of which 97 were excluded as they were duplicates, non‐relevant articles, reviews, opinions, letters to the editor or for another reason (e.g. unavailable or missing data) (Figure 1). Overall, six prospective cohort studies were included in the systematic review, reporting on a total of 10 408 patients^12^, ^13^, ^14^, ^15^, ^16^, ^17^. Of these 10 408 patients, 10 047 (96.5%) did not develop PE while 361 (3.5%) developed either preterm or term PE. The results of the quality assessment of the included studies using the QUADAS‐2 tool are presented in Table S1. The methodological characteristics of the included studies and the demographic characteristics of included patients are summarized in Table 1 and Table S2, respectively. Five studies^12^, ^14^, ^15^, ^16^, ^17^ investigated OA Doppler indices in an unselected general population with an undetermined risk for PE, who underwent standard antenatal ultrasound assessment in the first, second or third trimester of pregnancy. One study^13^ included women at high risk for PE, based on the presence of one or more PE risk factors. In two studies the OA was investigated in the first trimester, in three studies it was assessed in the second trimester and in one study it was assessed in the third trimester (Table 1). Among the OA Doppler parameters investigated and eventually implemented in the screening models, PSV2 was utilized in two studies^12^, ^13^ while PR was used in the remaining four studies^14^, ^15^, ^16^, ^17^, as the addition of these Doppler indices to traditional screening models demonstrated a significant improvement in the prediction of subsequent PE.

**Table 1:** Characteristics of studies reporting on the screening performance of ophthalmic artery (OA) Doppler indices for pre‐eclampsia (PE), included in systematic review

Study	Country	Study period	GA at assessment (weeks)	Study population	OA Doppler parameters assessed	Prevalence of PE (*n*/*N* (%))
Gurgel Alves (2014)^12^	Brazil	August 2009– March 2011	11–14	General population, with undetermined risk for PE	PI, PSV1, PSV2, PR	31/440 (7.0)
Matias (2014)^13^	Brazil	March 2010–June 2012	20–28	Women at high risk for PE (considering age at first pregnancy, history of PE, primipaternity or new father, multiple gestation, hypertension, diabetes, obesity)	PSV1, PSV2, EDV, PI, PR	40/347 (11.5)
Praciano de Souza (2018)^14^	Brazil	February 2011–October 2014	18–23	General population, with undetermined risk for PE	PI, PSV1, PR	40/415 (9.6)
Sarno (2020)^15^	UK	June 2019–March 2020	35 + 0 to 36 + 6	General population, with undetermined risk for PE	PI, PSV1, PSV2, PR	60/2287 (2.6)
Sapantzoglou (2021)^16^	UK	2019–2020	19 + 1 to 23 + 3	General population, with undetermined risk for PE	PI, PSV1, PSV2, PR	76/2853 (2.7)
Gana (2022)^17^	UK	June–August 2019 and May 2020–February 2021	11 + 0 to 13 + 6	General population, with undetermined risk for PE	PI, PSV1, PSV2, PR	114/4066 (2.8)

Only first author is given for each study. All studies were prospective and observational. EDV, end‐diastolic velocity; GA, gestational age; PI, pulsatility index; PR, ratio of second to first peak systolic velocity; PSV1, first peak systolic velocity; PSV2, second peak systolic velocity.

The definition of PE varied across studies: one study^13^ adopted the criteria of the Report of the National High Blood Pressure Education Program Working Group on High Blood Pressure in Pregnancy^18^, two studies^12^, ^14^ adopted the criteria determined by the International Society for the Study of Hypertension in Pregnancy in 2001^19^, and the rest of the included studies^15^, ^16^, ^17^ defined PE in accordance with the American College of Obstetricians and Gynecologists' Task Force on Hypertension in Pregnancy^20^.

### Synthesis of results

#### Maternal factors + ophthalmic artery Doppler

The pooled AUC of maternal factors in the prediction of overall PE was 0.71 (95% CI, 0.68–0.75). The combination of maternal factors with OA Doppler parameters had a pooled AUC of 0.79 (95% CI, 0.72–0.87) (Table 2). For the prediction of patients who developed preterm PE, the combination of maternal factors with OA Doppler had a pooled AUC of 0.88 (95% CI, 0.82–0.94), while the corresponding pooled AUC for those who developed term PE was 0.76 (95% CI, 0.65–0.88).

**Table 2:** Pooled area under the summary receiver‐operating‐characteristics curve (AUC) analyses of ophthalmic artery (OA) Doppler indices for prediction of pre‐eclampsia (PE)

Method of screening	Studies (*n* ^refs^)	Events/total (*n*/*N*)	Pooled AUC (95% CI)		*I* ^2^ (%)
			FEM	REM	
Maternal factors					
Overall PE	4^13^, ^14^, ^15^, ^17^	254/7032	0.71 (0.69–0.74)	0.71 (0.68–0.75)	0
Maternal factors + OA Doppler					
Overall PE	4^12^, ^13^, ^15^, ^17^	245/5948	0.79 (0.77–0.82)	0.79 (0.72–0.87)	72
Preterm PE	2^16^, ^17^	43/6772	0.88 (0.82–0.94)	0.88 (0.82–0.94)	0
Term PE	2^16^, ^17^	147/6876	0.76 (0.73–0.80)	0.76 (0.65–0.88)	89
Maternal factors + UtA‐PI					
Overall PE	4^12^, ^13^, ^15^, ^16^	207/5910	0.77 (0.74–0.80)	0.76 (0.66–0.88)	83
Preterm PE	2^16^, ^17^	43/6772	0.84 (0.78–0.91)	0.84 (0.78–0.91)	0
Term PE	2^16^, ^17^	147/6876	0.70 (0.66–0.74)	0.70 (0.62–0.78)	70
Maternal factors + UtA‐PI + OA Doppler					
Overall PE	3^12^, ^13^, ^15^	131/3057	0.85 (0.82–0.88)	0.85 (0.76–0.94)	54
Preterm PE	2^16^, ^17^	43/6772	0.88 (0.82–0.93)	0.88 (0.82–0.93)	0
Term PE	2^16^, ^17^	147/6876	0.78 (0.74–0.82)	0.77 (0.70–0.85)	0
Maternal factors + UtA‐PI + MAP					
Overall PE	3^14^, ^15^, ^16^	176/5489	0.85 (0.82–0.89)	0.80 (0.61–1.06)	79
Maternal factors + UtA‐PI + MAP + OA Doppler					
Overall PE	3^14^, ^15^, ^16^	176/5489	0.89 (0.86–0.92)	0.84 (0.61–1.15)	81

FEM, fixed‐effects model; MAP, mean arterial pressure; PI, pulsatility index; REM, random‐effects model; UtA, uterine artery.

#### Maternal factors + uterine artery Doppler + ophthalmic artery Doppler

The pooled AUC of the combination of maternal factors and UtA‐PI in predicting PE was 0.76 (95% CI, 0.66–0.88). The combination of maternal factors, UtA‐PI and OA Doppler had a pooled AUC of 0.85 (95% CI, 0.76–0.94) (Table 2). For the prediction of patients who developed preterm PE, the combination of maternal factors and UtA‐PI had a pooled AUC of 0.84 (95% CI, 0.78–0.91), while the corresponding figure for women who developed term PE was 0.70 (95% CI, 0.62–0.78). The combination of maternal factors and UtA‐PI with OA Doppler had a pooled AUC of 0.88 (95% CI, 0.82–0.93) for the prediction of preterm PE, while the corresponding figure for term PE was 0.77 (95% CI, 0.70–0.85).

#### Maternal factors + uterine artery Doppler + mean arterial pressure + ophthalmic artery Doppler

The pooled AUC of the combination of maternal factors, UtA‐PI and MAP in predicting PE was 0.80 (95% CI, 0.61–1.06) (Table 2). The combination of maternal factors, UtA‐PI and MAP with OA Doppler had a pooled AUC of 0.84 (95% CI, 0.61–1.15).

#### Maternal factors + mean arterial pressure + ophthalmic artery Doppler

The combination of maternal factors and MAP was omitted from this analysis, as their combination with OA was investigated in only two studies, one assessing the predictive value in the first trimester and the other in the third trimester.

## DISCUSSION

The prediction of preterm and term PE is the focal point of a growing area of investigation. It offers the potential for improved maternal and perinatal outcomes through intensified clinical monitoring and early administration of preventive medication, as there is mounting evidence supporting the efficacy of low‐dose aspirin in reducing the occurrence of adverse pregnancy outcomes^3^, ^21^, ^22^. Historically, the prediction of PE has relied on identifying clinical risk factors, with extensive research demonstrating a correlation between such indicators and an increased likelihood of developing PE^23^. Owing to the moderate accuracy of these models in predicting PE, recent research has concentrated on developing novel markers that can enhance the predictive accuracy, either independently or in combination with other biochemical or biophysical markers, in singleton and twin pregnancies^24^.

On this basis, two recent meta‐analyses performed by de Melo *et al*.^25^ and Dai *et al*.^26^ demonstrated significant differences in several OA Doppler indices between pre‐eclamptic and normotensive women. Specifically, the study of de Melo *et al*.^25^, which included 1425 patients, revealed an increase in the OA‐PR of normotensive women and an increase in the OA‐PSV2 of pre‐eclamptic women, while the meta‐analysis of Dai *et al*.^26^ demonstrated a significant decrease in OA‐PI and an increase in OA‐EDV in the group diagnosed with PE. Our study aimed to update the previously published meta‐analysis of Kalafat *et al*.^10^, as further studies investigating the added value of OA Doppler parameters in combination with already established combinations of biophysical markers (maternal factors alone, maternal factors combined with UtA‐PI, and maternal factors combined with UtA‐PI and MAP) have been published over the past years. Kalafat *et al*.^10^ included a total of three studies and 1119 patients, while our study included six studies and a total of 10 408 patients. As data accumulated, we were also able to group cases according to term or preterm PE, as the consequences and pathophysiology of the two types of PE may differ. As such, according to our meta‐analysis, the pooled AUC for the prediction of PE increased from 0.71 (95% CI, 0.68–0.75) with maternal factors alone to 0.79 (95% CI, 0.72–0.87) when OA Doppler was combined with maternal factors, although the overlapping 95% CIs suggest that the difference between the predictive models might not be significant. The discrimination was better in cases that developed preterm PE (AUC, 0.88 (95% CI, 0.82–0.94)) compared to those that developed term PE (AUC, 0.76 (95% CI, 0.65–0.88)). Similarly, the AUC for the development of term and preterm PE increased from 0.70 (95% CI, 0.62–0.78) and 0.84 (95% CI, 0.78–0.91), respectively, for the combination of maternal factors and UtA‐PI, to 0.77 (95% CI, 0.70–0.85) and 0.88 (95% CI, 0.82–0.93), respectively, when OA Doppler was combined with maternal factors and UtA‐PI, demonstrating a lesser impact of OA Doppler when UtA‐PI is already included. These findings support the theory of maternal cardiovascular system maladaptation in the development of subsequent PE, as has been suggested by several authors^27^, ^28^, ^29^, and Doppler assessment of the OA appears to be a simple, precise and objective tool for investigating these underlying alterations. However, as mentioned above, although direct comparisons between the models under investigation could not be performed, the overlapping 95% CIs indicate a potential lack of substantial statistical significance; these findings necessitate further evaluation.

Doppler assessment of the OA in pregnant women was initially suggested by Hata *et al*.^7^ in 1992. Further research validated a reduction in ophthalmic vascular resistance and an augmentation in perfusion among patients diagnosed with PE, showing either a lowering of the OA‐PI and resistance index (RI)^7^ or an increase in the PR^30^, ^31^, therefore demonstrating alterations to the maternal intracranial vascular circulation. Cerebrovascular changes as predictive markers for PE have been described previously in the studies of Belfort *et al*.^32^ and Williams and Moutquin^33^, which revealed that patients who later develop PE have lower values of maternal middle cerebral artery Doppler indices (RI and PI), indicating an early decrease in cerebrovascular impedance among women who will subsequently develop PE. This finding is supported by the studies included in our meta‐analysis, in which maternal OA Doppler indices demonstrated similar alterations.

The most prominent underlying causative pathophysiologic mechanisms of PE are thought to be impaired trophoblast development and defective conversion of the UtA and spiral arteries^34^. As such, predictive models for PE mostly rely on biophysical and biochemical indices that serve as markers for placentation and placental function, such as placental growth factor and UtA Doppler parameters^35^, ^36^, ^37^. However, several studies have provided evidence for a second theory that associates PE with elevated cardiac output, increased peripheral vascular resistance and a mild reduction in left ventricular systolic function, leading to a hyperdynamic circulatory state prior to the onset of clinically overt PE^1^, ^38^, ^39^, ^40^. The OA Doppler alterations demonstrated in our study are most likely explained by the aforementioned maternal hemodynamic changes, rather than trophoblast maldevelopment. Of note, the greater effect on the cardiovascular system associated with preterm PE compared with that of term PE could explain the more pronounced effect demonstrated by OA Doppler in the prediction of preterm PE when assessment takes place in the first or second trimester.

The main strength of our meta‐analysis is the inclusion of all available evidence on the predictive value of OA Doppler in combination with maternal factors and several biophysical markers for the subsequent development of term or preterm PE. We conducted an analytic search including six major prospective studies with no language restrictions and it should be highlighted that, despite the low number of included studies, a reasonable number of women (*n* = 10 408) were included in our analysis. We acknowledge that the present meta‐analysis is subject to several limitations, and several parameters may have contributed to these. Although direct comparisons could not be performed in any of the investigated comparison models (models with *vs* those without the addition of OA Doppler), the overlapping 95% CIs suggest a potential lack of significant difference. However, further evaluation is necessary in future studies using complementary tests, such as the DeLong test, which cannot be performed in synthetic studies that provide SROC curves. The timing of assessment varied among the included studies, with most conducting screening > 20 weeks' gestation while only two studies examined this novel Doppler parameter in the first trimester, which poses a concern as to the generalization of the results derived from first‐trimester assessment. As a consequence, this meta‐analysis is limited by the methodological heterogeneity of the included studies. More specifically, none of the included studies provided data concerning the actual false‐positive and false‐negative rates and the sensitivity and specificity of the provided algorithms. Hence, pooled AUCs were based on precalculated AUCs given in the included studies, thereby precluding the actual determination of the sensitivity and specificity of the addition of OA Doppler to the predictive algorithms. This limited the possibility of additional analyses, including the production of a Fagan's nomogram, which is essential for the determination of the post‐probability likelihood ratio of developing PE following the addition of OA Doppler, as well as a graphical representation of the hierarchical SROC curve, which would allow evaluation of the dispersion and heterogeneity of the provided data. Furthermore, it should be noted that only one study was performed in a population that was at intermediate‐to‐high risk for the development of PE, highlighting the variation in study design among the different research groups. Since the included studies span a study period of 12 years, the definition of PE varied across studies, with the adaptation of different diagnostic criteria possibly influencing the reported results. Therefore, further studies focusing on all pregnancy trimesters and aimed at both preterm and term PE are needed to ascertain the generalizability of the findings. Furthermore, prospective studies conducted in high‐risk populations, such as those with pre‐existing diabetes or hypertension, a previously affected pregnancy or those that have already developed pregnancy‐induced hypertension, are needed to assess the added value of OA Doppler in the prediction of subsequent PE in these patient categories.

To conclude, OA Doppler assessment is a fast and straightforward, yet precise, method for evaluating the underlying, less accessible intracranial circulation. The basic, standard equipment required for its evaluation supports its potential usefulness in low‐resource settings. The present meta‐analysis revealed that the addition of OA Doppler may increase the predictive performance for PE and, more precisely, for preterm PE, compared with models not involving this marker. However, the overlapping 95% CIs indicate a potential lack of significant differences between the different models, limiting the robustness and applicability of these results. Considering these limitations, our findings dictate the need for further research in both low‐risk and intermediate‐to‐high‐risk populations, focusing on the prediction of preterm PE, in which maternal and neonatal morbidity rates still constitute a major health issue, in order to ascertain and potentially expand the generalization of our results.

## Supporting information


**Table S1** Quality assessment of included studies using Quality Assessment of Diagnostic Accuracy Studies‐2 (QUADAS‐2) tool.

**Table S2** Demographic characteristics of patients included in systematic review.

## Data Availability

The data that support the findings of this study are available from the corresponding author upon reasonable request.
