# Supplementary material for: Added value of ophthalmic artery Doppler in prediction of pre‐eclampsia: systematic review and meta‐analysis
Source: Ultrasound Obstet Gynecol. 2025 Aug 19;66(6):716–23. doi: 10.1002/uog.70002 (PMC12671936; doi:10.1002/uog.70002)
Supplement: Supplementary file 1 — Table S1 Quality assessment of included studies using Quality Assessment of Diagnostic Accuracy Studies‐2 (QUADAS‐2) tool. [file UOG-66-716-s001.docx]

| Author, Year | Was a consecutive or random  sample of patients enrolled? | Was a case-control design  avoided? | Did the study avoid  inappropriate exclusions? | Risk of bias | Concerns regarding applicability | Were the index test results interpreted  without knowledge of the results  of the reference standard? | If a threshold was used,  was it pre-specified? | Risk of bias | Concerns regarding applicability | Is the reference standard likely to  correctly classify the target condition? | Were the reference standard results interpreted  without knowledge of the results  of the index test? | Risk of bias | Concerns regarding applicability | Was there an appropriate  interval between index test(s)  and reference standard? | Did all patients receive  a reference standard? | Did patients receive the  same reference standard? | Were all patients  included in the analysis? | Risk of bias |
| --- | --- | --- | --- | --- | --- | --- | --- | --- | --- | --- | --- | --- | --- | --- | --- | --- | --- | --- |
| **Gurgel Alves et al, 2014** | yes | yes | yes | low | low | yes | yes | low | low | yes | yes | low | low | yes | yes | yes | yes | low |
| **Matias et al, 2014** | no | yes | yes | unclear | low | yes | yes | low | low | yes | yes | low | low | yes | yes | yes | yes | low |
| **Praciano de Souza et al, 2018** | yes | yes | yes | low | unclear | yes | yes | low | low | yes | yes | low | low | yes | yes | yes | yes | low |
| **Sarno et al, 2020** | yes | yes | yes | low | low | yes | yes | low | low | yes | yes | low | low | yes | yes | yes | yes | low |
| **Sapantzoglou et al., 2021** | yes | yes | yes | low | low | yes | yes | low | low | yes | yes | low | low | yes | yes | yes | yes | low |
| **Gana et al, 2022** | yes | yes | yes | low | low | yes | yes | low | low | yes | yes | low | low | yes | yes | yes | yes | low |
