# Supplementary material for: Added value of ophthalmic artery Doppler in prediction of pre‐eclampsia: systematic review and meta‐analysis
Source: Ultrasound Obstet Gynecol. 2025 Aug 19;66(6):716–23. doi: 10.1002/uog.70002 (PMC12671936; doi:10.1002/uog.70002)
Supplement: Supplementary file 2 — Table S2 Demographic characteristics of patients included in systematic review. [file UOG-66-716-s002.docx]

| *Study* | ***Cases/****Controls* | Mean maternal age  (years) | Mean BMI (kg/m^2^) | Spontaneous  conception (%) | Nulliparity (%) | Smoking status (%) | Ethnicity (White/Black/Mixed) (%) | Prior hypertension (%) | Prior diabetes mellitus (%) | Personal history of PE (%) | Family history of PE(%) |
| --- | --- | --- | --- | --- | --- | --- | --- | --- | --- | --- | --- |
| Gurgel Alves (2014)^12^ | **31**/409 | **26.0**/26.1 | **28.2**/25.0 | N/R | **58.1**/49.1 | **0.0**/6.1 | **16.1/0.0/83.9**  23.0/2.9/74.1 | **9.7**/2.2 | **0.0**/2.9 | **32.2**/5.9 | **35.5**/14.7 |
| Matias (2014)^13^ | **40**/307 | **29.0**/24.0 | **27.7**/25.0 | N/R | **42.0**/54.0 | N/R | **7.0/93.0/N/R**  4.0/96.0/N/R | **23**/10 | **10.0**/3.0 | **23.0**/11.0 | **25.0**/23.0 |
| Praciano de Souza (2018)^14^ | **40**/309 | **27.0**/25.0 | **29.1**/25.2 | N/R | **52.5**/52.75 | **0.0**/4.2 | **25.0/5.0/65.0**  22.0/1.9/70.2 | N/R | N/R | **20.0**/7.11 | N/R |
| Sarno (2020)^15^ | **60**/2227 | **34.0**/33.6 | **30.6**/28.1 | **90.0**/94.6 | **81.7**/52.5 | **1.7**/0.4 | **75.0/16.7/3.3**  75.0/12.2/3.6 | **3.3**/1.5 | **3.3**/1.2 | **3.3**/1.9 | **8.3**/3.1 |
| Sapantzoglou (2021)^16^ | **76**/2777 | **34.9**/33.3 | **27.3**/25.4 | **79.0**/93.4 | **71.1**/53.5 | **0.0**/1.4 | **61.8/25.0/4.0**  74.2/13.5/3.7 | **9.2**/1.4 | **3.9**/1.0 | **13.2**/1.6 | **11.8**/2.9 |
| Gana (2022)^17^ | **114**/3952 | **32.1**/33.2 | **25.6**/24.3 | **92.1**/95.1 | **63.2**/51.1 | **0.9**/2.3 | **64.9/18.4/11.4**  74.6/12.9/3.4 | **6.1**/0.5 | **0.8**/0.7 | **10.5**/1.9 | **4.4**/2.9 |

**Table S2** Demographic characteristics of patients included in systematic review

Only first author is given for each study. The demographic characteristics of the cases are presented in **bold.** BMI, Body mass index; N/R, not reported; PE, pre-eclampsia.
